# Experimental Differentiation of Intraocular Masses Using Ultrahigh-Field Magnetic Resonance Imaging – A Case Series

**DOI:** 10.1371/journal.pone.0081284

**Published:** 2013-12-09

**Authors:** Karen Falke, Paul Krüger, Norbert Hosten, Annette Zimpfer, Rudolf Guthoff, Sönke Langner, Oliver Stachs

**Affiliations:** 1 Department of Ophthalmology, University of Rostock, Rostock, Germany; 2 Institute of Diagnostic Radiology and Neuroradiology, Greifswald University Hospital, Greifswald, Germany; 3 Institute of Pathology, University of Rostock, Rostock, Germany; Medical University Graz, Austria

## Abstract

**Purpose:**

The case reports presented here were compiled to demonstrate the potential for improved diagnosis and monitoring of disease progress of intraocular lesions using ultrahigh-field magnetic resonance microscopy (MRM) at 7.1 Tesla.

**Methods:**

High-resolution ex vivo ocular magnetic resonance (MR) images were acquired on an ultrahigh-field MR system (7.1 Tesla, ClinScan, Bruker BioScan, Germany) using a 2-channel coil with 4 coil elements and T2-weighted turbo spin echo (TSE) sequences of human eyes enucleated because of different intraocular lesions. Imaging parameters were: 40×40 mm field of view, 512×512 matrix, and 700 µm slice thickness. The results were correlated with in vivo ultrasound and histology of the enucleated eyes.

**Results:**

Imaging was performed in enucleated eyes with choroidal melanoma, malignant melanoma of iris and ciliary body with scleral perforation, ciliary body melanoma, intraocular metastasis of esophageal cancer, subretinal bleeding in the presence of perforated corneal ulcer, hemorrhagic choroidal detachment, and premature retinopathy with phthisis and ossification of bulbar structures. MR imaging allowed differentiation between solid and cystic tumor components. In case of hemorrhage, fluid-fluid levels were identified. Melanin and calcifications caused significant hypointensity. Microstructural features of eye lesions identified by MRM were confirmed by histology.

**Conclusion:**

This study demonstrates the potential of MRM for the visualization and differential diagnosis of intraocular lesions. At present, the narrow bore of the magnet still limits the use of this technology in humans in vivo. Further advances in ultrahigh-field MR imaging will permit visualization of tumor extent and evaluation of nonclassified intraocular structures in the near future.

## Introduction

The differential diagnosis of intraocular lesions is generally based on clinical findings supplemented by imaging. However, it is still a great challenge to image eye anatomy and pathology without artifacts. Ultrasound is the ophthalmologist's standard imaging tool for evaluating intraorbital structures [1], [2]. Other diagnostic techniques that can provide useful additional information include fluorescence angiography (FAG) [3], ultrasound biomicroscopy (UBM) [4], optical coherence tomography (OCT) [5], computed tomography (CT) [6], and magnetic resonance imaging (MRI) [7]. Nevertheless, a definitive diagnosis still requires histologic preparation [8].

With its excellent soft tissue contrast, MRI is well suited for the evaluation of orbital structures. MRI has already been used for imaging the posterior eye and orbit as well as the anterior segment [9]–[14]. Actually MRI techniques were not used for routine diagnostic of intraocular masses. Compared with standard clinical MRI scanners, MR microscopy (MRM) provides resolution in the sub-millimeter range while at the same time offering an excellent signal-to-noise ratio [15].

The aim of our study was to evaluate the potential of MRM in the diagnostic evaluation of intraocular masses and to compare the findings with routine diagnostic techniques and histology.

## Materials and Methods

### Patients

A total of 7 patients with clinically suspected intraocular masses were included in the case series. All patients ultimately required enucleation of the affected eye. Before routine histopathological work-up, all enucleated eyes were examined by ex vivo MRM. All patients gave written informed consent to participation and ex vivo MRM. This study was conducted after approval by the ethics review board of Greifswald University (7221.3-2.1-008/06).

### Clinical Imaging

Patients underwent clinical ultrasound (CineScan Quantel Medical, France) of the affected eye, and slit lamp photographs were taken for documentation. One patient (case 3) additionally underwent UBM (Vumax, Sonomed. Inc., USA). The affected eye of another patient (case 7) was additionally examined by clinical CT (Aquilion ONE, Toshiba, Germany) and MRI (1.5 T, Avanto, Siemens, Germany).

### Ex vivo MRM

The enucleated eyes were imaged on an ultrahigh-field MR scanner at 7.1 Tesla (ClinScan, Bruker Bioscan GmbH, Ettlingen, Germany) within 3 hours of enucleation. All eyes included were imaged using a phased-array transmit and receive surface coil with two channels and two coil elements for each channel. Eyes were placed on a gauze cushion inside the coil and imaged at room temperature. MRM was performed with a T2-weighted turbo spin echo sequence (T2w TSE). A field of view (FoV) of 40×40 mm with a matrix size of 512×512 pixels, interpolated to 1024×1024, was used. The other imaging parameters were: repetition time (TR) 3300 ms, echo time (TE) 75 ms, and turbo factor (TF) of 7. Twenty-two slices with no gap and a slice thickness of 700 µm were obtained. T2w images were acquired in three anatomical planes with an acquisition time of 8:16 min per plane. One eye was additionally imaged at 9.4 T (PharmScan, Bruker Biospin, Germany) using a helium-cooled cryocoil. T2w TSE images were acquired in axial and coronal plane.

### Histology

The enucleated eyes were fixed in 4% formalin. Macroscopically, 3 to 4 mm thick vertical sections from the lateral to the medial rectus muscle were cut after 2 days of fixation and embedded in paraffin. Subsequently, histological examination of the sections was performed using hematoxylin & eosin (H&E) staining. An attempt was made to obtain histological sections corresponding to MRM slice orientation.

## Results

**Figure 1:**
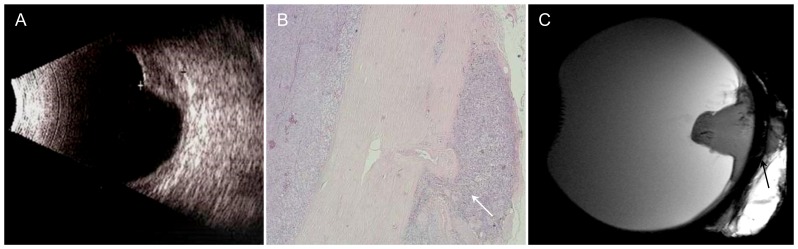
83-year-old patient with choroidal melanoma infiltrating the retrobulbar fat. A – Sonography: dome-shaped tumor of low reflectivity with a highly reflective surface. No information on extraocular tumor extension. B – H&E stain, 2× magnification: infiltration of the retrobulbar fat as demonstrated by MRM (arrow). C – Sagittal T2w image also demonstrates infiltration of the retrobulbar fat (arrow).

**Figure 2:**
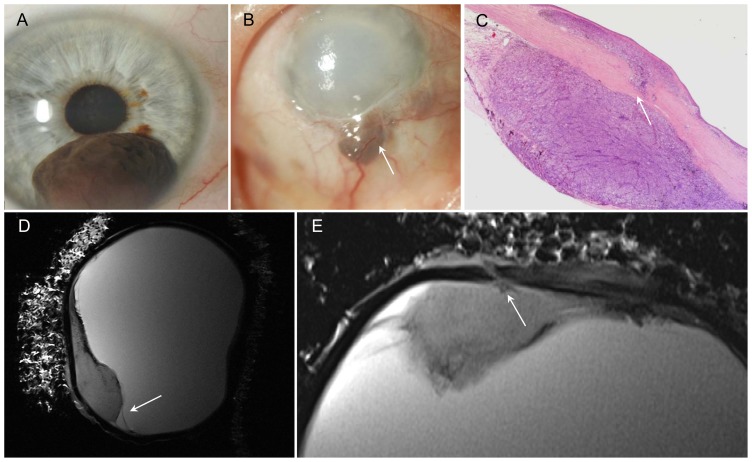
Malignant melanoma of iris and ciliary body with scleral perforation two years after iridocyclectomy. A – Photograph: malignant melanoma of iris at 6 o'clock before surgery (iridocyclectomy). B – Photograph: recurred tumor with scleral perforation at 6 o'clock, vascularization and corneal edema (arrow). C – H&E stain, 2× magnification: tumor extends through the sclera (arrow). Conjunctiva demonstrates mushroom-like detachment. D – Sagittal T2w image: tumor with hypointens rim (paramagnetic effect of melanin) and retinal detachment (arrow). E – Axial T2w image: delineation of the melanoma from the bulbar wall and good visualization of the perforation (arrow).

**Figure 3:**
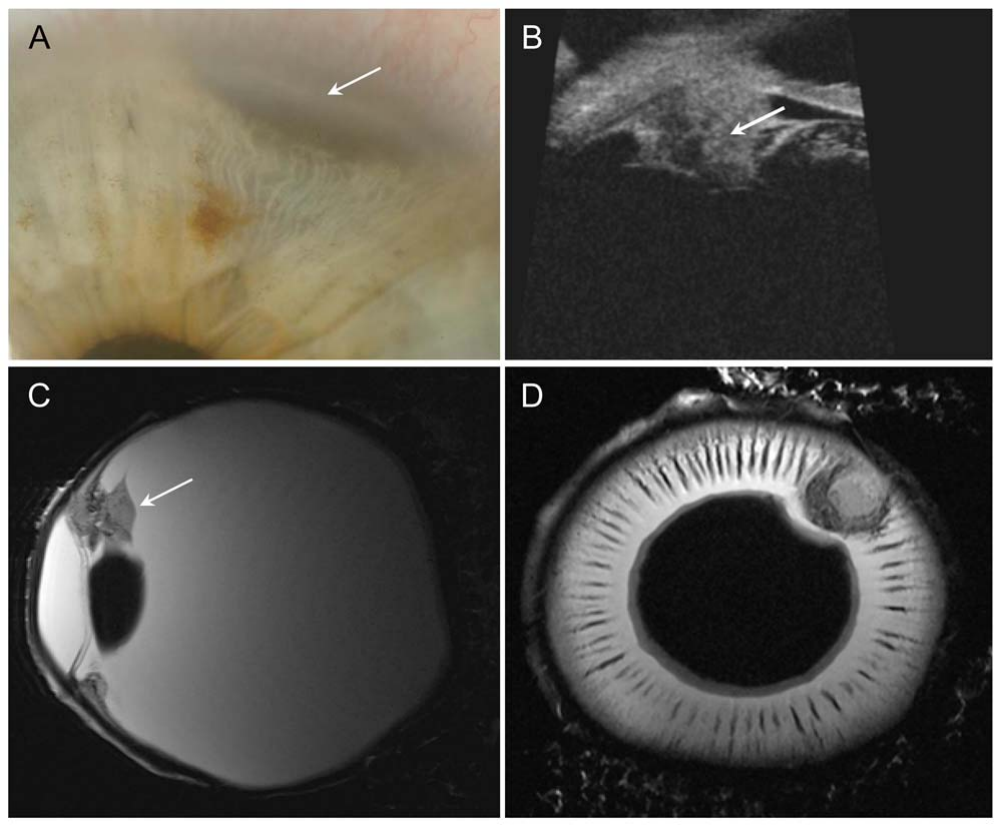
Ciliary body melanoma. A – Photograph: iris with visible melanoma at 1 o'clock (arrow). B – UBM of patient after ruthenium-106 plaque brachytherapy: residual tumor mass with height of 2.7 mm (arrow). C+D – Sagittal and axial T2w images: Inhomogeneous tumor invading the ciliary body (arrow). Low-signal-intensity cap-shaped mass representing the melanin-producing portion of the tumor. No scleral invasion.

**Figure 4:**
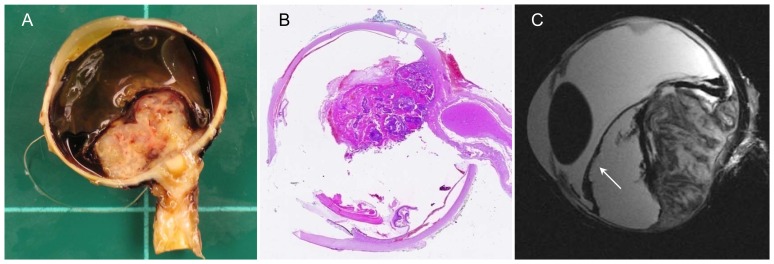
Intraocular metastasis of esophageal cancer with choroidal and retinal detachment (arrow). A – Enucleated eye with optic nerve segment. B – H&E stain, overview: A large, cloudy, solid tumor with retinal and choroidal detachment is apparent around the optic disc. C – Sagittal T2w image: Heterogeneous signal intensity reflecting the heterogeneous ultrastructure of the tumor with low-signal-intensity foci corresponding to intratumoral hemorrhage.

**Figure 5:**
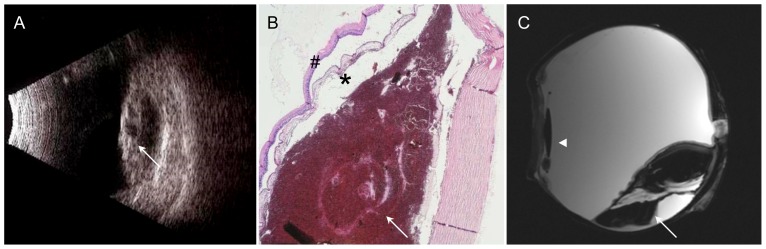
Subretinal bleeding with degenerative, atrophic choroid and retina. A – Sonography: highly reflective and detached cystic retina and choroidea (arrow); subretinal space with low internal reflectivity. B – H&E stain, 2× magnification: Peripapillary subretinal mass (arrow) with degenerative and atrophic lesions of the retina (hash) and choroid (asterisk). C – Sagittal T2w image: Subretinal mass of very low signal intensity with retinal detachment, fluid-fluid level (arrow), pseudophakia (arrowhead).

**Figure 6:**
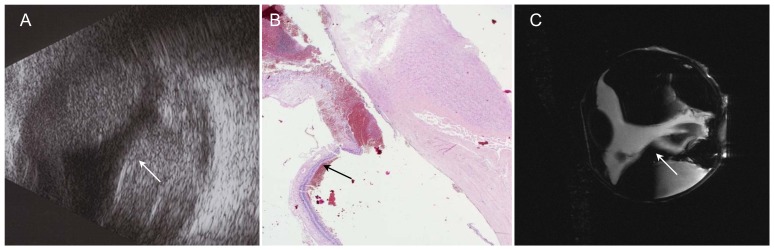
Hemorrhagic choroidal detachment (arrow). A – Sonography: Kissing bullae with high internal reflectivity. B – H&E stain, 2x: Portions of detached retina and choroid with blood underneath. C – Coronal T2w image: Very low internal signal intensity due to the presence of hemoglobin degradation products, fluid-fluid level in the lateral compartment.

**Figure 7:**
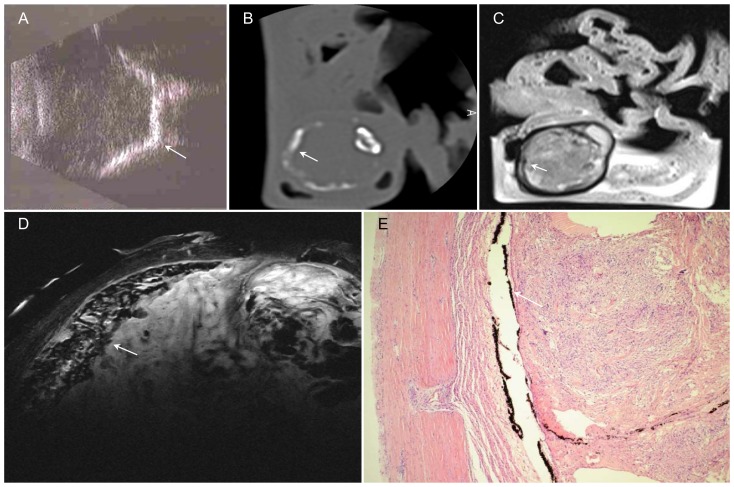
Premature retinopathy with phthisis and calcification of ocular wall (arrow). A – Sonography: Hyperreflective line corresponding to ossification process of bulbar wall. B – CT reconstructed in the bone window: corresponding plane to the MR image; the gross calcifications appear as linear hyperdensities. C – T2w image at 1.5 T: Bulbar wall with very low signal intensity; the vitreous body is of inhomogeneous low signal intensity, and ultrastructures cannot be identified with certainty. D – Sagittal T2w image: Thickened retina with very low signal intensity as a sign of calcification; intraocular scar tissue is characterized by inhomogeneous MR signal intensity. E – H&E stain, 4× magnification: Histologically, the intraocular space is filled with fibromatous scar tissue, and there is ossification of the bulbar wall originating in the pigment epithelium.

Seven enucleated eyes with the following pathologies were examined by MRM: choroidal melanoma (Fig 1), malignant melanoma of iris and ciliary body with scleral perforation (Fig. 2), ciliary body melanoma (Fig. 3), intraocular metastasis of esophageal cancer (Fig. 4), subretinal bleeding in the presence of perforated corneal ulcer (Fig. 5), hemorrhagic choroidal detachment (Fig. 6), and premature retinopathy with phthisis and ossification of bulbar structures (Fig. 7). The ex vivo MRM findings obtained in each of these pathologies are presented below.

### Choroidal melanoma (Fig. 1)

Enucleation was indicated because of tumor progression despite treatment. The patient initially underwent brachytherapy with a ruthenium-106 ophthalmic plaque (BEBIG GmbH, Germany, CGD applicator; dose rate of 6.83 Gy/h, calculated dose to sclera: 943 Gy, dose to apex: 161 Gy) and eight sessions of transpupillary thermotherapy; however, the tumor continued to grow, finally extending to the temporal margin of the optic disc. Ultrasound showed a dome-shaped mass of low reflectivity with a highly reflective surface. The tumor height was 4.1 mm, and the basal diameter was 11.8 mm. In this case ultrasound does not allow assessment of extraocular growth. Histopathology revealed a malignant choroidal melanoma of the epithelioid type with continuous, mass-like extension into the sclera and early extraocular invasion of retrobulbar fat in the area of the posterior pole. The melanoma was characterized by moderate melanin pigmentation, focal fibrosis, and residual older necrosis. T2-weighted MR microscopy images showed the dome shape of the tumor with tiny hypointensities resulting from the paramagnetic effect of melanin. There was excellent demarcation of small tumor foci extending into the retrobulbar fat and good correlation with histology.

### Malignant melanoma of iris and ciliary body with scleral perforation (Fig. 2)

In the case investigated, the patient had a spindle-cell malignant melanoma of the iris involving the ciliary body and the anterior chamber (height 4.8 mm, basal diameter 6.8). Two years after iridocyclectomy, the patient suffered tumor recurrence in the blind eye. Clinically, a pigmented tumor at the 6 o'clock position with extension through the sclera and local dilation of epiretinal vessels was diagnosed. Histological work-up demonstrated marked destruction of the residual ciliary body. The tumor extended through the sclera and reached the conjunctiva, which showed early signs of mushroom-like detachment. MR microscopy images in the sagittal plane allowed accurate evaluation of the true tumor extent and of retinal detachment. The tumor appeared ill defined with a small hypointense rim adjacent to the vitreous body on T2-weighted images, which was due to the paramagnetic effect of melanin. The axial image clearly delineated the site of perforation with good correlation to histology.

### Ciliary body melanoma (Fig. 3):

This patient had a malignant melanoma of the ciliary body seen in the 1 to 2 o'clock position. Following ruthenium-106 plaque brachytherapy (BEBIG GmbH, Germany, CCA applicator, dose rate of 4.94 Gy/h, calculated dose to sclera: 682 Gy, dose to apex: 173 Gy), there were residual tumor masses (height, 2.7 mm; basal diameter, 3.2). Over time, the patient experienced tumor progression and increasing pain because of secondary glaucoma. UBM showed cone-shaped residual tumor 2.7 mm in height after ruthenium-106 plaque brachytherapy. Tumor growth was nodular without scleral invasion. Histologically, malignant melanoma of the spindle cell type with segmental ciliary body involvement and severe depigmentation was diagnosed. T2-weighted sagittal and axial images showed an inhomogeneous tumor invading the ciliary body in the 2 o'clock position. The melanin-producing portion was seen as a low-signal-intensity cap-shaped mass with intraocular extension but without scleral invasion. There was good correlation of MRM with UBM and histology.

### Intraocular metastasis (Fig. 4)

The excised specimen included the eye and a short segment of the optic nerve. A large, cloudy, solid tumor with retinal and choroidal detachment was apparent around the optic disc. The histologic diagnosis was metastasis from esophageal adenocarcinoma. There were atypical gland-like epithelial proliferations with solid and papillary components around the site of entry of the optic nerve. The axial T2-weighted image showed the detached retina and choroid. MRM allowed evaluation of ultrastructure, again with good correlation to histology. The low-signal-intensity foci on T2-weighted images corresponded to intratumoral hemorrhage, seen both macroscopically and microscopically.

### Subretinal hemorrhage (Fig. 5)

In this case, enucleation was performed because of a perforated corneal ulcer. The eye was pseudophakic. Ultrasound revealed an intraocular, subchoroidal mass close to the optic nerve. Sonographically, the subretinal space had low internal reflectivity with a thickened, highly reflective detached retina and choroidea. Histology demonstrated a more recent hemorrhage presenting as a peripapillary subretinal mass with degenerative and atrophic lesions of the retina and choroid. T2-weighted images showed a subretinal mass of very low signal intensity with retinal detachment. There was a fluid-fluid level, which was predominantly located in the lateral compartment. The MRM appearance was characterized by low and intermediate signal intensities, reflecting a history of multiple serial hemorrhages with the presence of blood degradation products of different ages. The posterior chamber lens including the delicate lens haptic was depicted without geometric distortion.

### Hemorrhagic choroidal detachment (Fig. 6)

The patient underwent enucleation of a blind eye because of painful secondary glaucoma and hemorrhagic choroidal detachment. Ultrasound demonstrated bullous choroidal detachment and kissing bullae with high internal reflectivity. Histology confirmed these findings, showing portions of detached retina and choroid with blood underneath. On MRM, the appearance was characterized by very low internal signal intensity due to the presence of hemoglobin degradation products. A fluid-fluid level was seen in the lateral compartments. The lens appeared to be displaced inferiorly, suggesting involvement of lens supporting structures. There was very good correlation between MRM and ultrasound with respect to the distribution of hemorrhage. However, MRM allowed better and undistorted evaluation of the bulbar wall and other intraocular structures.

### Retinopathy of prematurity (Fig. 7)

In this case, enucleation was performed because of a painful hypotonic, blind eye due to retinopathy of prematurity. Clinically, there was atrophy of the bulb with complete internal organization. Sonographically, ossificationof the bulbar wall was suggested by a hyperreflective line. The clinical CT scan of the removed eye showed corresponding circumferential calcification of the bulbar wall. On clinical T2-weighted 1.5 T MR images, the bulbar wall had very low signal intensity. There was good correlation of these abnormal signal intensities with the changes seen on CT and sonography. The vitreous body was of inhomogeneous low signal intensity, and ultrastructures could not be identified with certainty. MR microscopy at 9.4 T allowed differentiation of the bulbar wall layers. The retina was thickened and, as a result of calcification, had very low signal intensity. Histologically, the intraocular space was filled with fibromatous scar tissue. Additionally, there was ossification of the bulbar wall originating in the pigment epithelium. The histologically proven scar tissue was characterized by inhomogeneous MR signal intensity. The histologic ultrastructure was reflected in the MR appearance.

## Discussion

The cases presented have been selected to illustrate the diagnostic potential of MRM. Compared with other imaging modalities used in ophthalmology, MRM visualizes lesion-specific features and allows undistorted and operator-independent assessment of eye anatomy and intraocular lesions. MRM correlates very well with histology in assessing solid tumor ultrastructure and identifying features such as hemorrhage or melanin deposits. Furthermore, MRM allows evaluation of extraocular tumor growth. It is difficult to detect extraocular growth using ultrasound [16]. In the cases presented, MRM was superior to ultrasound in demonstrating tumor invasion of the bulbar wall and infiltration of the vitrous body. MRM findings in the eyes with hemorrhagic retinal detachment demonstrate the ability of MRM to differentiate hemorrhage from other underlying causes of retinal detachment by the demonstration of a fluid-fluid level and to also evaluate the wall of the globe with its different layers underneath the hemorrhage. In preterm retinopathy and other conditions associated with solid transformation of the globe, MRM also allows assessment of solid and calcified areas with excellent spatial resolution.

Optical coherence tomography (OCT) allows non-contact high-resolution imaging of intraocular tumors [5], [17], [18]. OCT is widely available in the ophthalmic environment, and acquisition time is significantly shorter compared to MRM. For intraocular tumors, OCT provides valuable information regarding the status of the retina and the retinal pigment epithelium and can be useful in ascertaining reasons for visual loss. A disadvantage is that, as with all laser-based imaging techniques, the penetration depth is limited. Enhanced-depth OCT [17] is a new imaging modality that enables high-resolution OCT imaging of external retinal layers, the choroid, and lamina cribrosa. It reproducibly detects structural changes ‘beyond the RPE’ that are difficult to capture with standard OCT. MRM provides information not only on choroidal infiltration with good correlation to conventional histology but also on the extent of extraocular growth and scleral involvement.

External beam radiotherapy and brachytherapy, using iodine-125 or ruthenium-106 plaques, are the standard of care for small to medium sized choroidal melanomas [19], [20]. Enucleation has to be considered in patients with tumor recurrence, large intraocular masses, localization close to the optic nerve, or extraocular infiltration. Possible extraocular infiltration can be evaluated by MRM with excellent correlation to conventional histology. Therefore, MRM might have the potential to prevent enucleation in selected patients in the future.

The major limitation of the case series presented is that the small diameter of the bore precludes MRM of the human eye in vivo. The potential of MRM for visualization of eye structures in vivo has been reported in studies of the anterior and posterior segments of the eye. Strenk et al. presented visual targets to patients to image accommodation processes on a 1.5 T clinical MR imager [21], [22]. Strategies to reduce eye motion artifacts due to involuntary eye movement in 1.5 T and 3 T MRI of the posterior eye segment range from retrobulbar anesthesia [23], [24], acquisition of images with the imaged eye taped close and with the non-imaged eye fixating a target [25], [26] or image acquisition during a “non-blink cycle” in which the subjects were asked to refrain from blinking for a defined period [27]. The protocol of Bert and Patz [25], [26] as well as the strategy of Berkowitz [27] were also used on a 7 T scanner [28]. Since we performed ex vivo MRM, motion artifacts were not a concern. Therefore, we could also use longer acquisitions than reported in the literature [28]. Currently, we are developing a clinical ultrahigh-field MR system tailored to human eye imaging in vivo [29].

Our ex vivo case series of ultrahigh-field MRM at 7.1 T demonstrates that this technique provides detailed images of the eye, adjacent structures, and intraocular tumors with good correlation to histology. With increasing availability of ultrahigh-field MR systems and improvements in coil design, the role of in vivo MRM in comparison to widely available ophthalmologic imaging modalities such as ultrasound, UBM, and OCT has to be determined in further in vivo studies.
